# Genome-wide characterization of microsatellite DNA in fishes: survey and analysis of their abundance and frequency in genome-specific regions

**DOI:** 10.1186/s12864-021-07752-6

**Published:** 2021-06-07

**Authors:** Yi Lei, Yu Zhou, Megan Price, Zhaobin Song

**Affiliations:** 1 Sichuan Key Laboratory of Conservation Biology on Endangered Wildlife, College of Life Sciences, Sichuan University, Chengdu, 610065 People’s Republic of China; 2 Key Laboratory of Bio-Resources and Eco-Environment of Ministry of Education, College of Life Sciences, Sichuan University, Chengdu, 610065 People’s Republic of China

**Keywords:** Microsatellites, Genomic regions, Distribution patterns, Fish species

## Abstract

**Background:**

Microsatellite repeats are ubiquitous in organism genomes and play an important role in the chromatin organization, regulation of gene activity, recombination and DNA replication. Although microsatellite distribution patterns have been studied in most phylogenetic lineages, they are unclear in fish species.

**Results:**

Here, we present the first systematic examination of microsatellite distribution in coding and non-coding regions of 14 fish genomes. Our study showed that the number and type of microsatellites displayed nonrandom distribution for both intragenic and intergenic regions, suggesting that they have potential roles in transcriptional or translational regulation and DNA replication slippage theories alone were insufficient to explain the distribution patterns. Our results showed that microsatellites are dominant in non-coding regions. The total number of microsatellites ranged from 78,378 to 1,012,084, and the relative density varied from 4925.76 bp/Mb to 25,401.97 bp/Mb. Overall, (A + T)-rich repeats were dominant. The dependence of repeat abundance on the length of the repeated unit (1–6 nt) showed a great similarity decrease, whereas more tri-nucleotide repeats were found in exonic regions than tetra-nucleotide repeats of most species. Moreover, the incidence of different repeated types appeared species- and genomic-specific. These results highlight potential mechanisms for maintaining microsatellite distribution, such as selective forces and mismatch repair systems.

**Conclusions:**

Our data could be beneficial for the studies of genome evolution and microsatellite DNA evolutionary dynamics, and facilitate the exploration of microsatellites structural, function, composition mode and molecular markers development in these species.

**Supplementary Information:**

The online version contains supplementary material available at 10.1186/s12864-021-07752-6.

## Background

Microsatellites, also termed as simple sequence repeats (SSRs), are short tandemly repeated sequences with 1-to-6 base pair (bp) motifs [1, 2]. They are ubiquitous and highly abundant in eukaryote, prokaryote and virus genomes [3–5], making up around 3% of the human genome [6]. Microsatellite instability is an important and unique form of mutation that is responsible for, or strongly implicated in, over 40 human neurological, neurodegenerative and neuromuscular disorders [7] and associations have also been observed in other complex diseases [2, 3, 8, 9]. Undoubtedly, microsatellites have attracted considerable attention due to their roles in the organization of chromosome structure, DNA recombination and replication, and gene expression and cell cycle dynamics [10].

Microsatellite analysis is used for a wide range of biological questions. Unique polymorphism of normal and disease-causing repeats can be used for disease diagnosis and prognosis [11–13]. Microsatellite repeats are advantageous as genetic markers due to their high polymorphism, informativeness and co-dominance, and have been used to construct quantitative trait loci (QTL) maps, genetic linkage maps [14–18] and DNA fingerprinting [19]. These features also provide the foundation for their successful application in other fundamental and applied fields of biology, including population and conservation genetics, genetic dissection of complex traits and marker-assisted breeding programs [10, 20–22].

Microsatellite content generally correlates positively with genome size [23–25]. The distribution of microsatellites exhibit different properties in genomes with different functionality [26–31], contradicting earlier studies stating that they are randomly distributed and simply represent “junk” DNA sequences [32]. Microsatellites are ubiquitously distributed across the entire genome, including protein-coding and non-coding regions [6, 33–35]. Previous studies have indicated that microsatellite occurrence differs significantly in coding and non-coding regions [36], and some microsatellite types were preferred and often common in genome-specific regions [26, 29]. Excessive microsatellite repeats occur in non-coding regions of eukaryotic organisms [37], whereas they are relatively rare in coding regions, ranging between 7 and 10% of higher plants [38, 39] and between 9 and 15% of vertebrates [40–42]. Meanwhile, multiple studies have demonstrated that the hotspots of microsatellite distribution may be related with various phenotypic traits [43, 44]. In the genome of *Saccharomyces cerevisiae* about 17% of genes contain microsatellite repeats in open reading frames (ORFs) [45, 46] and the repeats are specifically enriched in regulatory genes that encode transcription factors, DNA-RNA binding proteins and chromatin modifiers [47]. Microsatellite repeats in *cis*-regulatory elements and promoters, which frequently occur (e.g., ~ 25% promoters in yeast contain tandem repeats), regulate the process of gene expression [48, 49]. The (TTAGGG) n tracts constitute a substantial portion of the telomeric regions and are recognized by telomerase, which can be related to stability of chromosomes and nucleolus organizing regions [10, 50, 51].

xMicrosatellites are inherently unstable with high mutation rates from about 10^− 6^ to 10^− 2^ per locus per generation, resulting from DNA replication slippage [52, 53]. Mutation rates vary among microsatellite types (perfect, compound or interrupted), base composition of the repeat [54], repeat types (di-, tri- and tetranucleotide) [55, 56] and lengths [21], and heterozygosity [57, 58], but also among chromosome position, cell division, the GC content in flanking DNA and taxonomic groups [59–62]. Microsatellite instability has a strong influence on genomic microsatellite abundance and various functions and is explained by two mutually exclusive mutational mechanisms: (i) DNA replication slippage theory suggests that during DNA replication, the nascent and template strand realign out of register, and if DNA synthesis continues unabated on this molecule the repeat number of the microsatellite is altered [21, 63, 64]. The stability of the slipped structure has been maintained by hairpin, triplex, cruciform or quadruplex arrangement of DNA strands [65–69]. (ii) Unequal recombination theory assumes that large scale contractions and expansions of the repeat array involved the processes of DNA unequal recombination, including unequal crossing over and gene conversion [70], via a number of transposable elements, the best known are *Alu* and other short interspersed elements [64, 71]. Non-reciprocal recombination, random genetic drift and selective forces could have a significant effect on the accumulation of tandem-repetitive sequences in genomes [63, 65, 70].

So far, systematic research regarding microsatellite variation and characterization have been conducted on phylogenetic lineages, including humans [41], primates [72–74], plants and fungi [36, 75–82], and viruses [83, 84]. Yet microsatellite distribution patterns in fishes, an important branch of biological evolution, remained unclear. Here, 14 fish genomes have been used to indicate the microsatellite distribution patterns. The main objectives of the present study were to examine the distribution patterns of microsatellite in different fish genomes. The specific aims were 1) to examine the abundance and frequency of microsatellites in several important fish genomes, and 2) to compare the compositional differences of microsatellites in different taxa and genome-specific regions. We anticipate our study will provide foundational knowledge of microsatellite dynamics in fish species, helping us to better understand microsatellite distribution, and provide strong support for further exploration of genome structure and microsatellite functions.

## Materials and methods

### Genomic sequences

Genome sequences from 14 fish species, including model fishes (*Danio rerio, Oryzias latipes, Astyanax mexicanus*), commercial species (*Cyprinus carpio, Oncorhynchus mykiss, Oncorhynchus kisutch, Oreochromis niloticus, Ictalurus punctatus, Esox Lucius, Cynoglossus semilaevis,*), ornamental fishes (*Poecilia reticulate, Takifugu rubripes, Nothobranchius furzeri*), and “living fossil” fish species (*Lepisosteus oculatus*), were used in this study. Most genome sequences were downloaded from the Ensembl Genome Browser (Ensembl, Available online: http://asia.ensembl.org/index.html). The sequences of *Cyprinus carpio*, *Nothobranchius furzeri*, *Oncorhynchus kisutch*, and *Oncorhynchus mykiss* were obtained from the National Centre for Biotechnology Information (NCBI. Available online: http://www.ncbi.nlm.nih.gov/). We also obtained genome annotations to identify microsatellite locations in the genomes. The genomic (chromosomal) sequences that had complete genome annotations were included in this study. We filtered the unknown bases (Ns) in genome sequences using the Perl script and obtained the valid length of sequences for further analysis. The details of the genome sequences are listed in Table 1.

**Table 1:** Data source and genome sizes of the 14 fish species studied in the present study

Order	Family	Species	Abbreviation	Source	The number of chromosomes	Total length of sequences (Mbp)^a^	Total valid length of sequences (Mbp)^a^	Unknown bases (Ns) in sequences (%)
Characiformes	Characidae	*Astyanax mexicanus*	Amex	Ensemble	25	930.61	896.76	3.75
Cypriniformes	Cyprinidae	*Cyprinus carpio*	Ccar	NCBI	50	825.07	809.20	1.91
Cypriniformes	Cyprinidae	*Danio rerio*	Drer	Ensemble	25	1345.10	1340.61	0.33
Pleuronectiformes	Cynoglossidae	*Cynoglossus semilaevis*	Csem	Ensemble	22	445.14	424.00	5.28
Esociformes	Esocidae	*Esox lucius*	Eluc	Ensemble	24	796.47	787.67	1.12
Siluriformes	Ictaluridae	*Ictalurus punctatus*	Ipun	Ensemble	29	762.01	752.24	1.29
Lepisosteiformes	Lepisosteidae	*Lepisosteus oculatus*	Locu	Ensemble	29	891.14	834.92	9.31
Cyprinodontiformes	Nothobranchiidae	*Nothobranchius furzeri*	Nfur	NCBI	19	1078.72	715.84	32.96
Beloniformes	Adrianichthyidae	*Oryzias latipes*	Olat	Ensemble	24	723.44	582.14	19.51
Salmoniformes	Salmonidae	*Oncorhynchus kisutch*	Okis	NCBI	30	1686.58	1628.60	3.43
Salmoniformes	Salmonidae	*Oncorhynchus mykiss*	Omyk	NCBI	29	1949.96	1754.52	10.09
Cichliformes	Cichlidae	*Oreochromis niloticus*	Onil	Ensemble	23	868.59	864.36	0.49
Cyprinodontiformes	Poeciliidae	*Poecilia reticulata*	Pret	Ensemble	23	696.70	637.75	8.43
Tetraodontiformes	Tetraodontidae	*Takifugu rubripes*	Trub	Ensemble	22	281.57	268.85	4.64

^a^(Mbp) megabase pair

### Microsatellite identification

Microsatellites were identified from genome sequences using the Krait v0.9.0 program, a robust and ultrafast tool with a user-friendly graphic interface for genome-wide investigation of microsatellites [85]. We employed the perfect search model of the program to investigate the all motifs according to minimum repeats or minimum length of microsatellite. In the present study, we defined the perfect microsatellites as being mononucleotide repeats ≥12-bp, dinucleotide repeats ≥14-bp, trinucleotide repeats ≥15-bp, tetranucleotide repeats ≥16-bp, pentanucleotide repeats ≥20-bp and hexanucleotidue repeats ≥24-bp, and the length of flanking sequence was constrained to 200 bp, as previously described [72, 74]. We mainly examined the distribution of perfect repeats ≥12-bp long. The rationale for choosing the small cutoff value was that the microsatellites are often disrupted by single base substitutions [6, 33]. The occurrence of repeats in exons, introns and intergenic regions have been identified from the annotations of the 14 fish genome sequences using Perl scripts. The SciRoKo software tool [86] and the NCBI Graphical Sequence Viewer program (https://www.ncbi.nlm.nih.gov/projects/sviewer/) were employed to increase the reliability of the results for examined microsatellite repeats.

Repeats with unit patterns being circular per-mutations and/or reverse complements of each other were grouped together as one type. The total number of the non-overlapping type was 501 for 1–6 nt long motifs, with 1-nt motif containing two 2 types: A and C (A = T and C = G), 2-nt motif containing 4 types: AT, AG, AC and GC (AT = TA, AG = GA = CT = TC, AC=CA = GT = TG, and GC=CG), and 3–6-nt motif containing 10, 33, 102 and 350 types [41, 87].

## Results

### Distribution patterns of microsatellite repeats in the fish genomes

We examined the number, relative frequency (microsatellite numbers per Mb of the sequence), relative density (total microsatellite length per Mb of the sequence), GC content and the coverage degree (percentage of total microsatellites length in sequence) of microsatellites with motif lengths of 1–6 nucleotides in the 14 fish genomes (Table 2). We assigned 4-letter name abbreviations to the 14 species and these have been henceforth used to simplify results and discussion (e.g. *Danio rerio* = Drer, *O. niloticus* = Onil; see Table 2). The total number of microsatellites, ranging from 78,378 (Locu) to 1,012,084 (Drer), differed between fish species and the coverage degree varied from 0.18% (Locu) to 5.29% (Trub) (Table 2). The lowest relative frequency and relative density of microsatellites were both found in Olat (249.99 loci/Mb and 4925.76 bp/Mb, respectively) (Table 2). The highest relative frequency and density of microsatellites was found in Csem (3445.94 loci/Mb) and Ipun (25,401.97 bp/Mb), respectively. The GC content ranged from 10.94% (Trub) to 48.20% (Okis) (Table 2).

**Table 2:** Microsatellite distribution as frequency, density and GC content of different fish genomes

Repeat type	Species	Abbreviation	Total numbers	Total length (bp)	Relative abundace (loci/Mb)	Relative Density (bp/Mb)	Coverage degree (%)	G + C	
								Length (bp)	content (%)
Mono- (1-nt)	*D. rerio*	Drer	207,951	3,278,888	155.12	2445.81	0.24	156,865	4.78
	*O. niloticus*	Onil	85,303	1,316,072	98.69	1522.59	0.15	139,335	10.59
	*O. latipes*	Olat	76,805	1,120,875	131.94	1925.44	0.19	198,155	17.68
	*T. rubripes*	Ttub	34,748	504,976	129.25	1878.28	0.19	264,888	52.46
	*I. punctatus*	Ipun	176,856	2,991,388	237.37	4013.86	0.40	343,854	11.49
	*C. semilaevis*	Csem	97,303	1,483,637	232.06	3539.85	0.35	231,161	15.58
	*E. lucius*	Eluc	45,139	667,053	62.88	928.80	0.08	326,439	48.94
	*O. mykiss*	Omyk	90,887	1,336,990	51.64	759.16	0.08	690,373	51.64
	*N. furzeri*	Nfur	50,594	805,921	71.06	1131.40	0.11	364,314	45.20
	*L. oculatus*	Locu	26,639	347,118	39.34	517.74	0.04	80,197	23.10
	*A. mexicanus*	Amex	170,345	3,398,972	189.44	3753.31	0.38	266,645	7.84
	*C. carpio*	Ccar	186,935	2,883,267	230.83	3558.04	0.37	84,049	2.91
	*P. reticulata*	Pret	119,330	1,775,344	188.83	2810.96	0.23	66,159	3.73
	*O. kisutch*	Okis	96,581	1,542,599	59.64	952.88	0.09	993,999	64.44
Di- (2-nt)	*D. rerio*	Drer	382,761	14,766,368	285.51	11,014.63	1.10	3,485,035	23.60
	*O. niloticus*	Onil	123,095	3,294,102	142.41	3811.02	0.38	1,337,732	40.61
	*O. latipes*	Olat	25,449	515,736	43.72	885.93	0.09	203,111	39.38
	*T. rubripes*	Trub	63,408	12,729,440	235.85	6432.72	4.73	837,010	6.58
	*I. punctatus*	Ipun	359,022	10,686,166	484.03	14,362.27	1.42	3,777,407	35.35
	*C. semilaevis*	Csem	127,607	2,668,806	2998.81	6238.79	0.63	1,071,149	40.14
	*E. lucius*	Eluc	150,812	3,215,024	212.80	4533.65	0.41	1,466,606	45.62
	*O. mykiss*	Omyk	390,181	11,517,856	225.44	7684.48	0.66	5,186,111	45.03
	*N. furzeri*	Nfur	186,289	6,543,766	261.89	9175.17	0.91	3,065,853	46.85
	*L. oculatus*	Locu	16,853	289,242	30.03	527.85	0.03	122,671	42.41
	*A. mexicanus*	Amex	446,448	12,194,636	498.90	13,549.68	1.36	3,706,651	30.40
	*C. carpio*	Ccar	181,614	5,801,364	224.78	7173.27	0.72	2,057,824	35.47
	*P. reticulata*	Pret	70,237	1,810,180	110.24	2843.81	0.28	858,108	47.40
	*O. kisutch*	Okis	502,660	12,625,074	310.50	7784.45	0.78	6,023,864	47.71
Tri- (3-nt)	*D. rerio*	Drer	140,175	3,374,835	104.56	2517.38	0.25	262,890	7.79
	*O. niloticus*	Onil	33,649	662,808	38.93	766.82	0.08	166,799	25.17
	*O. latipes*	Olat	15,732	295,236	27.02	507.16	0.05	104,498	35.39
	*T. rubripes*	Ttub	12,671	262,275	47.13	975.54	0.10	117,321	44.73
	*I. punctatus*	Ipun	91,842	2,466,039	123.29	3302.90	0.32	324,772	13.17
	*C. semilaevis*	Csem	48,581	1,052,241	114.46	2478.47	0.25	408,981	38.87
	*E. lucius*	Eluc	11,632	208,425	15.88	284.64	0.03	62,534	30.03
	*O. mykiss*	Omyk	37,290	704,511	21.39	404.12	0.05	210,431	29.87
	*N. furzeri*	Nfur	39,742	918,591	55.80	1288.97	0.13	221,669	24.13
	*L. oculatus*	Locu	26,590	644,715	35.80	822.92	0.08	93,309	14.47
	*A. mexicanus*	Amex	61,775	2,572,995	69.16	2833.21	0.29	333,193	12.95
	*C. carpio*	Ccar	53,774	1,273,065	66.86	1588.10	0.16	127,770	10.04
	*P. reticulata*	Pret	24,815	616,578	39.01	966.24	0.10	144,894	23.50
	*O. kisutch*	Okis	34,901	643,725	21.72	401.60	0.04	213,491	33.16
Tetra- (4-nt)	*D. rerio*	Drer	243,740	8,195,596	181.81	6113.32	0.61	2,326,652	28.39
	*O. niloticus*	Onil	32,971	731,160	38.14	845.90	0.08	210,529	28.79
	*O. latipes*	Olat	22,084	776,628	37.94	1334.10	0.13	250,470	32.25
	*T. rubripes*	Trub	16,187	541,848	60.21	2015.43	0.20	231,326	42.69
	*I. punctatus*	Ipun	108,518	2,422,148	145.59	3347.91	0.32	526,093	21.72
	*C. semilaevis*	Csem	34,100	768,504	79.96	1807.35	0.18	277,262	36.08
	*E. lucius*	Eluc	21,952	411,372	30.05	562.93	0.05	191,704	46.60
	*O. mykiss*	Omyk	70,423	1,850,584	40.46	1062.57	0.11	826,372	44.65
	*N. furzeri*	Nfur	55,909	1,531,664	78.34	2142.29	0.21	530,688	34.65
	*L. oculatus*	Locu	4439	92,396	7.76	153.69	0.01	35,673	38.61
	*A. mexicanus*	Amex	84,721	3,160,820	95.30	3525.04	0.35	884,624	27.99
	*C. carpio*	Ccar	62,915	1,749,772	77.78	2162.41	0.22	374,219	21.39
	*P. reticulata*	Pret	32,055	1,138,844	50.22	1777.93	0.18	421,839	37.04
	*O. kisutch*	Okis	90,546	2,527,184	55.65	1551.12	0.16	1,163,069	46.02
Penta- (5-nt)	*D. rerio*	Drer	35,377	1,419,895	26.39	1059.14	0.11	104,811	7.38
	*O. niloticus*	Onil	12,089	316,950	13.99	366.69	0.04	69,140	21.81
	*O. latipes*	Olat	4973	145,115	8.54	249.28	0.02	36,644	25.25
	*T. rubripes*	Ttub	3871	164,760	14.40	612.83	0.06	87,874	53.33
	*I. punctatus*	Ipun	9556	234,630	12.84	315.11	0.03	50,793	21.65
	*C. semilaevis*	Csem	5828	186,075	13.57	428.73	0.04	54,885	29.50
	*E. lucius*	Eluc	1140	26,200	1.55	35.77	0.00	11,874	45.32
	*O. mykiss*	Omyk	13,985	342,135	7.96	194.80	0.02	122,566	35.82
	*N. furzeri*	Nfur	10,079	275,340	14.04	382.28	0.04	76,366	27.74
	*L. oculatus*	Locu	2292	56,690	3.10	73.65	0.01	20,527	36.21
	*A. mexicanus*	Amex	16,919	1,094,190	18.90	1187.90	0.12	282,448	25.81
	*C. carpio*	Ccar	15,817	686,070	19.57	850.94	0.08	79,829	11.64
	*P. reticulata*	Pret	3815	125,295	5.97	195.30	0.02	32,651	26.06
	*O. kisutch*	Okis	17,343	428,935	10.64	263.32	0.03	166,246	38.76
Hexa- (6-nt)	*D. rerio*	Drer	2080	58,950	1.55	43.97	0.00	19,507	33.09
	*O. niloticus*	Onil	966	26,856	1.12	31.07	0.00	8832	32.89
	*O. latipes*	Olat	485	13,884	0.83	23.85	0.00	5484	39.50
	*T. rubripes*	Trub	1127	34,446	4.19	128.12	0.01	18,573	53.92
	*I. punctatus*	Ipun	1671	44,820	2.24	59.92	0.01	15,581	34.76
	*C. semilaevis*	Csem	2996	106,962	7.08	252.92	0.03	50,107	48.85
	*E. lucius*	Eluc	712	23,232	1.01	33.21	0.00	11,962	51.49
	*O. mykiss*	Omyk	4590	140,352	2.66	81.33	0.01	71,447	50.91
	*N. furzeri*	Nfur	1664	44,028	2.33	61.70	0.01	20,000	45.42
	*L. oculatus*	Locu	1565	53,070	1.84	62.81	0.01	23,037	43.41
	*A. mexicanus*	Amex	2179	193,794	2.43	209.55	0.02	60,384	31.16
	*C. carpio*	Ccar	1724	69,300	2.13	88.33	0.01	20,736	29.92
	*P. reticulata*	Pret	786	21,594	1.23	33.73	0.00	11,193	51.83
	*O. kisutch*	Okis	5178	169,488	3.20	104.70	0.01	85,527	50.46
Total	*D. rerio*	Drer	1,012,084	31,094,532	754.94	23,194.25	2.31	6,355,760	20.44
	*O. niloticus*	Onil	288,073	6,347,948	333.28	7344.09	0.73	1,932,367	30.44
	*O. latipes*	Olat	145,528	2,867,474	249.99	4925.76	0.48	798,362	27.84
	*T. rubripes*	Trub	132,012	14,237,745	491.03	12,042.92	5.29	1,556,992	10.94
	*I. punctatus*	Ipun	747,465	18,845,191	1005.36	25,401.97	2.50	5,038,500	26.74
	*C. semilaevis*	Csem	316,415	6,266,225	3445.94	14,746.11	1.48	2,093,545	33.41
	*E. lucius*	Eluc	231,387	4,551,306	324.17	6379.00	0.57	2,071,119	45.51
	*O. mykiss*	Omyk	607,356	15,892,428	349.55	10,186.46	0.93	7,107,300	44.72
	*N. furzeri*	Nfur	344,277	10,119,310	483.46	14,181.81	1.41	4,278,890	42.28
	*L. oculatus*	Locu	78,378	1,483,231	117.87	2158.66	0.18	375,414	25.31
	*A. mexicanus*	Amex	782,387	22,615,407	874.13	25,058.69	2.52	5,533,945	24.47
	*C. carpio*	Ccar	502,779	12,462,838	621.95	15,421.09	1.56	2,744,427	22.02
	*P. reticulata*	Pret	251,038	5,487,835	395.50	8627.97	0.81	1,534,844	27.97
	*O. kisutch*	Okis	747,209	17,937,005	461.35	11,058.07	1.11	8,646,196	48.20

The main distribution pattern of di- (mononucleotide SSRs) > mono- (dinucleotide SSRs) > tetra- (trinucleotide SSRs) > tri- (tetranucleotide SSRs) > penta- (pentanucleotide SSRs) > hexanucleotide (hexanucleotide SSRs) was shared by six fish genomes (i.e., Drer, Trub, Ipun, Amex, Eluc, and Omyk), while a mono- > di- > tetra- > tri- > penta- > hexanucleotide pattern was observed in Olat, Ccar and Pret (Table 2). The di- > mono- > tri- > tetra- > penta- > hexanucleotide pattern was shared by Onil and Csem, whereas Nfur exhibited a di- > tetra- > mono- > tri- > penta- > hexanucleotide pattern (Table 2). The 1-nt or 2-nt repeats had a higher percentage motif abundance in the fish genomes than any other motif length, while the 2-nt repeats represented more than 60% motif abundance in Omyk, Eluc and Okis (Table 3). There was an almost equal distribution of motif abundance percentages between the first three motifs (1–3 nt) in Locu, with 3-nt and 1-nt repeats being almost identical (34.92, 34.99%, respectively). The percentage of 4-nt repeats was remarkably uniform across all taxa except for Drer and Locu, which had marginally greater or lesser percentages of this motif length (Table 3). Microsatellites with longer motifs (5–6 nt) showed lower percentages compared to the short motif repeats (1–4 nt). The 6-nt repeats had the lowest percentages among these motif lengths, ranging from 0.21% (Drer) to 0.85% (Trub) (Table 3).

**Table 3:** Microsatellite distribution as percent motif abundance (%) among 14 genomes

Species	Abbreviation	Genomic region	Mono- (1-nt)	Di- (2-nt)	Tri- (3-nt)	Tetra- (4-nt)	Penta- (5-nt)	Hexa- (6-nt)
*D. rerio*	Drer	all	20.54	37.82	13.85	24.08	3.50	0.21
		intergenic regions	2.10	5.66	1.79	3.42	0.40	0.02
		introns	18.06	31.55	11.72	20.54	3.10	0.18
		exons	0.39	0.60	0.34	0.12	0.01	0.00
*O. niloticus*	Onil	all	29.61	42.73	11.68	11.45	4.20	0.34
		intergenic regions	1.91	4.23	1.15	0.89	0.53	0.07
		introns	25.15	36.73	9.01	10.02	3.55	0.24
		exons	2.56	1.77	1.53	0.53	0.12	0.02
*O. latipes*	Olat	all	52.77	17.49	10.81	15.18	3.42	0.33
		intergenic regions	6.26	2.19	1.37	2.21	0.55	0.05
		introns	45.06	14.57	7.54	12.56	2.81	0.27
		exons	1.45	0.73	1.90	0.40	0.06	0.02
*T. rubripes*	Trub	all	26.32	48.03	9.60	12.26	2.93	0.85
		intergenic regions	7.19	13.91	2.79	4.15	1.11	0.28
		introns	17.07	32.71	5.40	7.81	1.78	0.52
		exons	2.06	1.42	1.42	0.30	0.04	0.05
*N. furzeri*	Nfur	all	14.71	54.15	11.55	16.2	2.91	0.48
		intergenic regions	4.53	17.11	3.83	5.66	1.13	0.16
		introns	9.87	37.05	7.25	10.79	1.85	0.34
		exons	0.57	1.25	0.73	0.24	0.04	0.01
*O.mykiss*	Omyk	all	14.91	64.33	6.12	11.59	2.29	0.75
		intergenic regions	0.26	1.27	0.24	0.3	0.11	0.04
		introns	14.17	61.7	5.27	11.22	2.18	0.7
		exons	0.57	1.27	0.63	0.24	0.02	0.02
*A. mexicanus*	Amex	all	21.75	57	7.29	10.88	2.17	0.28
		intergenic regions	1.05	3.26	0.53	0.77	0.16	0.04
		introns	20.03	52.99	6.84	9.89	1.97	0.23
		exons	0.68	0.81	0.52	0.17	0.03	0.01
*E. lucius*	Eluc	all	19.51	65.18	5.03	9.49	0.49	0.31
		intergenic regions	1.1	3.45	0.33	0.7	0.06	0.04
		introns	17.19	60.03	3.92	8.54	0.42	0.26
		exons	1.22	1.7	0.78	0.25	0.01	0.01
*I. punctatus*	Ipun	all	23.68	48	12.3	14.25	1.28	0.22
		intergenic regions	1.17	3.05	0.82	0.89	0.09	0.02
		introns	20.97	43.26	10.64	13.18	1.12	0.19
		exons	1.52	1.71	0.82	0.45	0.07	0.01
*C. semilaevis*	Csem	all	31.05	39.78	15.25	11.11	1.85	0.96
		intergenic regions	1.79	1.63	0.78	0.76	0.11	0.07
		introns	27.47	37.29	12.76	9.72	1.66	0.8
		exons	1.49	1.41	1.81	0.3	0.07	0.07
*L. oculatus*	Locu	all	34.99	21.5	34.92	5.66	2.93	2
		intergenic regions	6.57	4.81	7.46	1.26	0.73	0.51
		introns	24.71	15.27	23.44	4.06	2.05	1.36
		exons	2.71	1.42	3.01	0.35	0.14	0.13
*C. carpio*	Ccar	all	37.18	36.12	10.7	12.51	3.15	0.34
		intergenic regions	0.54	0.6	0.17	0.19	0.05	0.01
		introns	36.2	35.16	10.2	12.22	3.08	0.33
		exons	0.44	0.37	0.32	0.11	0.02	0.01
*P. reticulata*	Pret	all	46.3	29.54	9.69	12.69	1.48	0.31
		intergenic regions	2.25	2.01	0.59	0.1	0.12	0.03
		introns	40.47	26.13	7.47	11.34	1.27	0.24
		exons	3.58	1.39	1.63	0.36	0.09	0.04
*O.kisutch*	Okis	all	12.85	67.38	4.61	12.15	2.31	0.7
		intergenic regions	0.19	1.16	0.13	0.22	0.06	0.02
		introns	12.29	65.73	4.17	11.84	2.23	0.68
		exons	0.37	0.49	0.32	0.09	0.01	0.00

### Mononucleotide repeats

Motif abundance percentages of mononucleotide repeats within intergenic regions, introns and exons varied across species, with intergenic regions ranging from 0.19% (Okis) to 7.19% (Trub), introns ranging from 9.87% (Nfur) to 45.06% (Olat) and exons ranging from 0.37% (Okis) to 3.58% (Pret) (Table 3). Among the two types of mononucleotide repeats, poly(A/T) was generally far more abundant than poly(C/G) in these fish genomes, except that the reverse was found in the Trub, Omyk and Okis genome sequences (Supplement Table 1, Tables 4 and 5). Drer had the maximum repeat number of A (or T) (192,264) followed by Ccar, Ipun, Amex and Pret. Pret contained the maximum number (4549) of C (or G). Although poly(A/T) tracts were clearly more abundant than poly(C/G) in exons (Table 4), this difference was not consistently observed in introns (Table 5) and intergenic regions (Table 6).

**Table 4:** Total numbers of Mono-, Di-, and Trinucleotide repeats in exons among 14 fish genomes

Repeated unit	*O. latipes*	*D. rerio*	*O. niloticus*	*T. rubripes*	*E. lucius*	*L. oculatus*	*O. mykiss*	*C. carpio*	*I. punctatus*	*C. semilaevis*	*A. mexicanus*	*N. furzeri*	*P. reticulata*	*O. kisutch*
A	1938	3830	6968	2079	2069	1652	2766	2178	10,981	4478	5149	1376	9181	1453
C	177	105	397	643	758	359	686	57	364	232	207	471	242	1341
AC	746	4274	3272	1429	2858	493	4435	1179	8327	2870	3180	3345	2238	2205
AG	97	1021	1121	272	679	459	2236	457	3402	1273	3234	412	877	991
AT	196	787	683	174	382	156	1020	203	1010	314	824	237	525	535
CG	24	10	9	1	5	4	5	3	68	3	14	39	12	–
AAC	145	332	435	86	172	182	365	171	850	437	150	136	252	206
AAG	235	346	542	154	151	108	434	153	680	587	385	178	641	274
AAT	170	775	665	118	179	156	350	309	2124	338	729	314	544	191
ACC	152	142	249	126	186	114	377	69	199	296	236	145	185	262
ACG	382	289	591	274	102	291	247	209	343	1159	528	417	704	184
ACT	14	111	133	30	43	20	62	46	123	148	84	265	66	51
AGC	322	172	239	203	102	255	202	113	199	519	614	55	474	124
AGG	1031	574	1141	591	576	777	1245	220	853	1731	856	46	923	782
ATC	199	660	353	121	235	93	495	321	726	397	401	752	306	304
CCG	120	32	51	165	48	247	27	19	64	129	84	14	194	11

**Table 5:** Total numbers of Mono-, Di-, and Trinucleotide repeats in introns among 14 fish genomes

Repeated unit	*O. latipes*	*D. rerio*	*O. niloticus*	*T. rubripes*	*E. lucius*	*L. oculatus*	*O. mykiss*	*C. carpio*	*I. punctatus*	*C. semilaevis*	*A. mexicanus*	*N. furzeri*	*P. reticulata*	*O. kisutch*
A	54,469	168,546	65,095	10,417	20,201	13,989	41,783	176,323	142,517	74,854	143,668	17,966	102,376	34,546
C	11,110	8113	7349	12,124	19,575	3979	44,066	5666	14,260	12,080	13,067	13,704	4070	58,278
AC	14,896	151,933	72,301	36,853	107,530	6473	219,574	102,632	180,297	71,964	142,073	99,236	48,930	25,072
AG	1311	16,700	12,758	4675	19,458	3157	116,077	26,618	78,058	20,922	112,135	11,308	9216	195,437
AT	4937	129,210	20,624	1546	11,767	2307	38,935	47,442	64,634	25,056	160,352	8320	10,458	35,958
CG	55	255	135	100	145	33	162	69	392	63	58	322	134	113
AAC	896	9018	4035	543	796	1633	3550	5763	10,973	4836	1646	146	1911	2654
AAG	762	2806	2699	350	173	393	1874	2606	5733	4440	4239	3781	2253	1683
AAT	4610	88,156	11,327	1362	3558	11,224	10,690	37,268	45,617	10,492	31,950	8898	9074	8967
ACC	539	677	941	448	921	764	1297	293	1217	783	1134	964	394	1954
ACG	706	508	1153	692	96	264	681	589	428	5941	2032	1125	1183	940
ACT	115	3189	858	354	652	226	2025	1107	3542	1476	2492	4481	388	2846
AGC	517	296	511	749	69	223	715	288	242	2754	2028	1291	926	542
AGG	1671	1427	2156	1359	679	694	3286	630	1678	6136	2180	78	1747	4327
ATC	1005	8440	2186	929	2092	975	7870	2708	10,070	3209	5727	1278	1605	7564
CCG	151	64	77	337	38	113	17	46	61	303	121	35	162	17

**Table 6:** Total numbers of Mono-, Di-, and Trinucleotide repeats in intergenic regions among 14 fish genomes

Repeated unit	*O. latipes*	*D. rerio*	*O. niloticus*	*T. rubripes*	*E. lucius*	*L. oculatus*	*O. mykiss*	*C. carpio*	*I. punctatus*	*C. semilaevis*	*A. mexicanus*	*N. furzeri*	*P. reticulata*	*O. kisutch*
A	7053	19,888	4667	4118	1523	3883	846	2603	7815	4738	7386	8467	5671	515
C	2058	1337	827	5367	1013	1170	740	108	919	921	867	7139	237	907
AC	2238	32,637	7840	15,342	5880	2113	3698	1873	13,324	2766	8393	48,648	3896	4585
AG	202	4756	1780	2175	1082	962	3056	445	6202	1023	7170	5467	705	3532
AT	741	19,891	2563	780	1020	683	979	693	3277	1346	9921	4626	684	606
CG	6	45	9	61	6	13	4	–	31	7	4	169	13	10
AAC	109	1260	438	255	49	537	190	79	866	281	92	797	133	64
AAG	117	476	296	167	19	146	70	51	500	376	291	2127	193	74
AAT	782	13,857	1526	673	369	4014	368	597	3336	621	2247	5103	699	170
ACC	74	95	65	250	58	230	30	5	87	30	104	566	25	28
ACG	160	103	131	421	6	68	5	16	44	373	108	576	90	68
ACT	22	214	52	290	48	86	197	26	298	84	269	837	25	234
AGC	167	57	74	335	2	42	12	5	16	173	144	39	65	8
AGG	349	364	345	653	70	224	187	11	183	340	175	734	143	197
ATC	180	214	367	467	143	326	422	56	782	162	715	2384	155	122
CCG	30	14	13	169	–	34	–	–	8	30	12	28	19	–

### Dinucleotide repeats

Among genome-specific regions, there was a lower percentage of dimer repeats (AT, AC, AG, CG) in exons compared to non-coding regions, ranging from 0.37% (Ccar) to 1.77% (Onil). Within the non-coding regions, intronic regions have a higher proportion of dinucleotide repeats compared to the intergenic regions (Table 3). We found that (AC) n repeats were generally more numerous in specific genomic regions, except that Amex had greater numbers of (AG) n repeats in exonic regions and Okis had greater (AT) n repeats in intronic regions (Tables 4 and 5). The number of (AT) n repeats observed the greatest variation between genome-specific regions and species. For example, intronic or intergenic regions of Drer have similar numbers of (AT) n repeats to (AG) n repeats, whereas exon numbers of (AT) n repeats were considerably less than (AG) n repeats. Olat had more (AT) n repeats than (AG) n in exons, but the opposite was found in other genomes. Finally, (CG) n repeats were very infrequent or absent in these genomes.

### Trinucleotide repeats

Motif abundance percentages of trinucleotide repeats in the exons of six fish species were greater than in intergenic regions, these six species being Olat (1.90%), Csem (1.81%), Pret (1.63%), Onil (1.53%) and Eluc (0.78%) (Table 3). Meanwhile, motif abundance percentages of trinucleotide repeats in the exons of Locu, Csem and Olat were greater than other motif lengths (e.g. mono-, di-). Among the different trinucleotide repeats, (AAT) n repeats were generally the most numerous repeats in intronic and intergenic regions of different taxa (Tables 5 and 6), except for Okis where (ACT) n repeats were the most numerous in intergenic regions (Table 6). There was no one trinucleotide repeat in exonic regions that was typically more numerous than another across the different fish species. For example, (AAT) n repeats were most numerous in Drer and Ipun, while (ATC) n repeats were greater in Ccar and Nfur and (AGG) n repeats were greatest in the 10 remaining species (Table 4). Repeats such as ACT, ACT, AGC, ACG and CCG were generally in low numbers in each specific genomic region. Furthermore, CCG repeats were absent in the intergenic regions of Eluc, Omyk, Ccar and Okis (Table 6).

### Tetranucleotide repeats

Tetranucleotide repeats were frequent in each genomic region and were generally dependent on the base composition of the repeat unit (Tables 7, 8, 9 and Supplement Table 1). Overall, repeats with > 50% of A + T (e.g. AAAT, ATAG and AATC repeats) were more abundant in studied fish genomes (Supplement Table 1). There were, however, a few notable exceptions. For example, (ACAG) n repeats were the most numerous in Eluc, Omyk and Okis (Supplement Table 1). We found that the (AAAB) n repeats (where B denotes any base other than A) were most numerous in exonic regions in five fish species (i.e. Olat, Drer, Onil, Ccar and Ipun), the (ACAG) n repeats were numerous in Eluc, Omyk and Okis, and (ATCC) n repeats were most common of the remaining four fish species (Table 7). Similar to exons, the most common tetranucleotide repeat in intergenic regions was (AAAB) n, except for (ATCC) n in Olat, (AATC) n in Eluc and (ATAG) n in Amex (Table 9). In introns, (AATB) n or (ACAG) n were the most common tetranucleotide repeats in studied fish (Table 8). We also found some repeats with > 50% of C + G (e.g. ACGC, AGGG and AGCG repeats) were in the top 50% of tetranucleotide repeats in specific genome regions (Tables 7, 8, 9).

**Table 7:** The most frequent Tetra-, Penta-, and Hexanucleotide repeats in introns^a^

Length of repeated unit	Taxa													
	*O. latipes*	*D. rerio*	*O. niloticus*	*T. rubripes*	*E. lucius*	*L. oculatus*	*O. mykiss*	*C. carpio*	*I. punctatus*	*C. semilaevis*	*A. mexicanus*	*N. furzeri*	*P. reticulata*	*O. kisutch*
4-nt	AAAT(4298)	AAAT(46819)	AAAT(7252)	ATCC(2657)	ACAG(4505)	AAAC(394)	ACAG(12845)	AAAT(26188)	AAAT(36182)	ATCC(7288)	ATAG(25367)	ATCC(7081)	ATCC(7303)	ACAG(30164)
	ATCC(4197)	ATAG(27227)	AAAC(4710)	ACAG(1263)	AGGG(2259)	ATCC(338)	AAAT(9010)	ATAG(11853)	AAAC(11498)	ATAG(4977)	AAAT(13454)	AAAT(5962)	AAAT(6468)	AGGG(9858)
	ATAG(1888)	ATCC(23910)	ATCC(4352)	ATAG(812)	AATC(2201)	AAAT(282)	AGGG(6350)		ATCC(10151)	AAAT(3814)		ACGC(4103)	ATGG(1988)	AAAT(9303
		AATG(22059)		AAAT(747)	ACGC(1493)	AATC(253)	ACGC(5380)					ATAG(3514)		
						ACAG(228)								
5-nt	AATTC(892)	AATAT(12313)	AATAG(2669)	AGAGG(1286)	ATACC(157)	AATTC(168)	AAATC(5447)	AAAAT(3745)	AAAAC(1282)	AGAGG(592)	AATGT(2997)	AATTC(1286)	AAAAT(580)	AAATC(7042)
	AAATC(552)	AAAAT(4838)	AATTC(2375)		AGAGG(59)	AAAAC(104)	AGAGG(1922)	AATAT(3347)	AATTC(1214)	AATCT(465)	AATAT(1814)	ATACC(775)	AAAAC(457)	AGAGG(4021)
	AATAG(494)		AAAAT(868)		ATACG(49)	AATAC(80)		AAATT(1092)	AATAT(1079)	AAAAT(383)	AAAAT(1119)	AATAG(684)	AATAG(326)	
	AAAAT(273)				AACGG(44)	AATAT(70)			AAAAT(965)	AATAT(346)	AAGAG(918)	AAAAT(327)	AATAT(244)	
					ACCCC(36)	AAATC(66)				AAAAC(326)	AATAG(535)		AAAAG(158)	
					AGGGG(35)	AAAAT(61)				ACTGG(270)	ATATC(496)			
					AAAAC(34)	ATGAC(60)				ATGTC(250)				
					ATCGC(29)	AAATT(41)								
					AACTG(28)	AAGCG(35)								
					AAGGC(28)	ACTGG(34)								
						CCCGG(34)								
6-nt	AATCAG(75)	AAATGT(244)	AATCAG(107)	AACCCT(67)	ACACGC(94)	AACTTG(150)	ACAGAG(730)	AAAAAT(282)	ATACTG(99)	ACCAGG(156)	AATCCC(216)	ACACGC(236)	AACCAG(236)	ACAGAG(1106)
	ATATAG(28)	ACACGC(234)	ATATAC(87)	AGAGGG(42)	AACCCT(78)	AACAGG(51)	ACACGG(468)	AAATGT(187)	AAATGT(96)	ACACAG(134)	AAACTG(93)	AATCAG(102)	ACACGC(53)	AGAGGG(472)
	AAATTC(18)	AAATAC(211)	ATATAG(52)	AATCCC(37)	ACACAG(45)	ACACTC(39)	AGAGGG(367)	AATCAG(174)	ATATAC(94)	AACCAG(101)	AAATGT(84)	AAGACG(71)	AAAAAT(26)	ACACGC(383)
	AACCCT(16)	ATATAC(113)	AAAAAC(43)	ACAGAG(32)	AATCCC(41)	ACACGC(34)	AACCCT(321)	AAAGTG(124)	ATAGTC(65)	ATATAC(94)	AAAAAT(61)	AAATAC(52)		ATACAC(279)
	AATCCC(12)	AAAAAT(89)	AAAAAT(30)	ACCCCC(24)	ATATAC(40)	AATAAG(31)	ACACAG(272)	AATCCC(92)	ATATAG(62)	AAGAGG(90)	AAATAT(53)	AAACAT(50)		AAAGAG(250)
	AAAAAT(10)		AATAAG(26)	AATCAG(21)	AGCGGG(39)	AATCTG(30)			AAATAT(60)	ACCTGG(77)	ATATAC(53)	ATACAG(34)		ACACAG(223)
	ATAGAG(10)		AAATTC(23)	ACACGC(21)		AAATAT(29)			AACCCT(57)	ACAGAG(69)	AATCGT(52)	AATCCC(32)		
	AAATAC(9)			AATCTG(20)		ACACAG(28)			AAAAAT(52)	AAATAT(68)	AATGCT(51)			
	AAAAAG(8)			AATTCT(18)		AATCCC(25)			AAAAAC(43)	AGAGGG(54)	ATATAG(45)			
	AAAATT(7)			ACTGGG(18)		AATAAC(21)			ATACAC(33)	AACCCT(49)	AAAGTC(44)			
				AAGAGG(17)		AACGGG(20)			AATCCC(32)	ACTCCC(48)	ACACTC(40)			
				ACACTC(17)		AACCCT(19)			AATAGT(27)	ACCTCC(45)	AAAGAG(38)			
				ACACAG(16)						AAAAAT(43)	ATGTCC(37)			
										AATCCC(43)	AAATCC(36)			
										ACTGGG(43)	AACCCT(34)			
										ATCAGC(39)				
										AAGATG(38)				
										AGTCGG(37)				
										ATGACC(37)				

^a^Only the repeat motifs that together comprise 50% of all repeats of the particular unit length are shown here

^b^Hexanucleotide repeats for which the number of the tandem repeat is < 2 are not shown

**Table 8:** The most frequent Tetra-, Penta-, and Hexanucleotide repeats in exons^a^

Length of repeated unit	Taxa													
	*O. latipes*	*D. rerio*	*O. niloticus*	*T. rubripes*	*E. lucius*	*L. oculatus*	*O. mykiss*	*C. carpio*	*I. punctatus*	*C. semilaevis*	*A. mexicanus*	*N. furzeri*	*P. reticulata*	*O. kisutch*
4-nt	AAAT(123)	AAAT(336)	AAAC(474)	AAAC(57)	ACAG(99)	AAAC(44)	ACAG(286)	AAAT(181)	AAAT(1063)	AAAC(245)	AAAT(264)	AAAT(212)	AAAT(231)	ACAG(117)
	AAAC(91)	AAAC(229)	AAAT(339)	AAAT(57)	ACGC(68)	AGGG(32)	AAAT(119)	AAAC(121)	AAAC(928)	AAAT(124)	AAAC(232)	AAAC(109)	AAAC(166)	AAAT(61)
	ATCC(65)	AATG(109)		ACGC(42)	AAAT(53)	AAAT(17)	ATAC(106)			AAAG(76)	AAAG(104)	ACGC(109)	AAAG(89)	ATAC(61)
	AAAG(39)			ACAG(39)	ATCG(41)	AGCG(16)	AGGG(97)			ACAG(66)	ACAG(95)			ATCG(53)
				AAAG(32)	ATAC(39)	ACGG(14)	ATCG(79)							ACTG(41)
						AAAG(13)	ACGC(76)							
5-nt	AAAAT(18)	AATAT(19)	AAAAC(79)	AAAAT(6)	ACTGC(2)	AAAAC(13)	AAATC(26)	AAAAC(13)	AAAAC(145)	AAAAC(30)	AAAAT(35)	AAAAT(26)	AAAAC(53)	AAATC(17)
	AATAG(8)	AAAAC(17)	AAAAT(49)	AAAAC(5)	AGCCC(2)	ACCGG(11)	AGAGG(25)	AAAAT(11)	AAAAT(67)	AGAGG(28)	AAAAC(25)	AATAG(13)	AAAAT(37)	AAAAC(6)
	AATTC(7)	AAAAT(13)	AAAAG(43)	AGAGG(5)	AGGGG(2)	CCCGG(9)	AATAC(7)	AATAT(7)	AAAAG(44)	AAAAG(24)	AATGT(20)	AATTC(11)	ACCGG(29)	AGAGG(6)
	AAAAG(5)	AAATT(7)		AAAAG(4)	ATCTC(2)	AGGCG(8)	AAAAC(5)	AACTG(4)	AATAT(20)	ACTGG(14)	AATAT(12)	AGAGG(8)	AAAAG(16)	AAAAT(4)
	AAACT(5)	AATCG(7)		CCCGG(4)	CCCCG(2)	AGAGG(5)	ACCCC(5)	AATAG(4)		AAAAT(13)	AAAAG(11)	AATGT(7)		AACAG(4)
				ACAGG(3)		AAAAT(4)		AGAGG(4)			AAGCT(11)			
				AAATT(2)		ACCCG(4)					AATCG(10)			
											AAGAG(9)			
6-nt	ACTCGG(3)	ACACGC(6)	AAAAAC(6)	ATACCG(8)	ACACCG(4)	AAGAGG(5)	AGAGGG(12)	AGCCGG(4)	AAAAAT(8)	ACCCAG(36)	ACGAGG(4)	ACACGC(5)	AACCAG(24)	AGAGGG(5)
	AACCAG(2)	ACACTC(3)	AAAAAT(3)	ACCAGG(3)	ACACGC(4)	ACTCCC(5)	ACAGAG(7)	AAGAGG(2)	AAAAAC(6)	AACCAG(12)	AAAAAT(3)	AATCAG(3)	ACCTGG(6)	AAAACC(3)
	AAGATG(2)	AAGATG(2)	AACCAG(3)	ACGAGG(3)	AACCAG(2)	AGCCGG(5)	AAGAGG(5)	ACAGGG(2)	AAAAAG(6)	ACCTGG(12)	AAGAGG(3)	AAAAAC(2)	AAGAGG(5)	AACCAC(2)
	ATGTCC(2)	ACCTCC(2)	AAAAAG(2)	ACGGGG(3)	ACCGAG(2)	ACGAGG(4)	ACACGC(5)	ACCCCG(2)	AAGAGG(4)	ATCAGC(12)	AATCGT(2)	AACCAG(2)	ACCTCC(4)	AACCCT(2)
	etc.^b^	AGCCCG(2)	AAGAGG(2)	ACTCGG(3)	ACCTCC(2)	AGAGGG(4)	ACTCCC(4)	ACGCAG(2)	ATATAC(4)	ACCTCC(9)	ACACTC(2)	AAGAGG(2)	ATCTGG(4)	AAGAGG(2)
		etc.^b^	ACCCGG(2)	AGCCGG(3)	AGGCGG(2)	ACCCGG(3)	AGGGGG(3)	ATCCTC(2)	ACACCC(3)	AAGAGG(8)	ACCAGG(2)	AGCTCC(2)	ACGCCG(3)	AGCTCC(2)
			ACTCCG(2)	ATCCTC(3)		ACCGGG(3)	AAAAAT(2)		ACCCCC(3)	AGCAGG(8)	ACCTCC(2)		ACTCCG(3)	
			AGCTCC(2)	AACACC(2)		ACGGGG(3)	AAAGAG(2)		ACGAGG(3)	ACAGAG(7)	ACTCTC(2)		AGCGGC(3)	
			ATGCCC(2)	AAGGAG(2)		AGTCGG(3)	AACACC(2)		AGGGGG(3)	ACCACG(5)	AGTCCG(2)		AATACT(2)	
				AATCCC(2)		ATGCCC(3)	AACCCT(2)		ATACAC(3)	ACGAGG(5)				
				AATCTG(2)		AACAGC(2)	ACCCAG(2)		ATCCTC(3)	ACTGGG(5)				
				ACAGAG(2)		ACACTC(2)	ACCGCC(2)			ATCCTC(5)				
						ACCCCC(2)				AAAAAC(5)				
						ACCGGC(2)								
						AGCCCC(2)								

^a^Only the repeat motifs that together comprise 50% of all repeats of the particular unit length are shown here

^b^Hexanucleotide repeats for which the number of the tandem repeat is < 2 are not shown

**Table 9:** The most frequent Tetra-, Penta-, and Hexanucleotide repeats in intergenic regions^a^

Length of repeated unit	Taxa													
	*O. latipes*	*D. rerio*	*O. niloticus*	*T. rubripes*	*E. lucius*	*L. oculatus*	*O. mykiss*	*C. carpio*	*I. punctatus*	*C. semilaevis*	*A. mexicanus*	*N. furzeri*	*P. reticulata*	*O. kisutch*
4-nt	ATCC(889)	AAAT(7753)	AAAT(505)	ATCC(1513)	AATC(327)	AAAC(153)	ACAG(453)	AAAT(418)	AAAT(2306)	ATAG(657)	ATAG(2538)	ATCC(4513)	ATCC(787)	ACAG(600)
	AAAT(597)	ATAG(4886)	ATCC(468)	ATAG(527)	ACAG(295)	AAAT(112)	AAAT(246)	ATAG(147)	AAAC(794)	ATCC(525)	AAAT(778)	AAAT(3225)	AAAT(477)	AGGG(237)
	ATAG(347)	AATG(4285)	AAAC(431)	ACAG(502)	ATCC(169)	ATCC(108)	AGGG(167)		ATAG(790)	AAAT(199)		ATAG(2429)		
		ATCC(4223)		AAAT(380)	ATAC(111)	AATC(106)	ACTG(134)							
						ACGC(51)								
5-nt	AATTC(203)	AATAT(1421)	AATTC(466)	AGAGG(865)	ATACC(27)	AATTC(83)	AAATC(209)	AAAAT(61)	AATTC(81)	ACTGG(47)	AATGT(176)	AATCC(866)	AAAAT(43)	AGAGG(167)
	AAATC(119)	AAAAT(601)	AATAG(384)		ATACG(15)	AATAC(41)	AGAGG(25)	AATAT(37)	AAAAC(75)	AATAT(31)	AATAT(98)	ATACC(540)	AATAG(30)	AAATC(155)
	AATAG(86)	AAATT(405)			AACAC(8)	AAAAC(39)		AATTC(19)	AATAT(71)	AAAAT(27)	ATATG(96)	AATAG(533)	AAAAC(22)	
					AAGGC(7)	AATAT(29)		AATAG(16)	AAAAT(63)	AACTG(26)	AAAAT(74)		AAAAG(21)	
					AATGC(7)	AAATC(25)			ATCAC(41)	ATGTC(25)	AATAG(73)		AATAT(20)	
					AACGG(6)	AAAAT(24)			AAAAG(35)	AATCT(21)	AAGAG(62)		ACCGG(15)	
						AATAG(17)					AAACC(60)		AGAGG(11)	
						ACTGG(16)								
						ATGAC(16)								
6-nt	AATCAG(12)	AAATGT(33)	AATCCC(113)	AACCCT(51)	AACCCT(16)	AACTTG(56)	AACCCT(74)	AAAGTG(6)	AACCCT(43)	ACCAGG(18)	AACCCT(136)	ACACGC(95)	AACCAG(40)	ACAGAG(31)
	ACCTGG(4)	ACACGC(33)		AATCCC(43)	AATCCC(10)	AACAGG(33)	ACAGAG(37)	AAAAAT(5)	AATCCC(21)	AACCAG(15)	AATCCC(47)	AATCAG(74)	ACACGC(9)	AACCCT(18)
	AAACTG(3)	AAATAC(27)		AATCTG(16)	ACACGC(7)	AAATAT(17)	AATCCC(31)	AATCCC(5)	ATCCAC(8)	ACCTGG(14)		AAGACG(43)		ACACAG(18)
	AACCCT(3)	ATATAC(13)		AATCAG(15)	ACACAG(5)	ACACTG(17)			AAATGT(6)	AAGTCG(12)		AAATAC(41)		ACACGG(9)
	AAGAGG(3)	AATCCC(12)		ACCCCC(15)	ACTCTC(5)	AATCTG(13)			ATATAC(6)	ATCAGC(12)		ATACAG(30)		
	AATCCC(3)	AAAAAT(11)		ACAGAG(14)		ACACGC(10)				ACACAG(11)				
	ATCTGG(3)			AGAGGG(14)		AATAAG(9)				ATGACC(11)				
	AAATTG(2)			AAGAGG(10)		ACACTC(9)				AAAAAT(7)				
	AACCAG(2)			AATTAG(10)						ACACTG(7)				
										AAGATG(6)				
										ACTGGG(6)				

^a^Only the repeat motifs that together comprise 50% of all repeats of the particular unit length are shown here

^b^Hexanucleotide repeats for which the number of the tandem repeat is < 2 are not shown

### Pentanucleotide repeats

As expected, the occurrence pentanucleotide repeats was less than tetranucoeitde repeats in different genome regions. We found a general distribution pattern of pentanucleotide repeats for all species, where (A + T)-rich repeats were the most abundant. Yet, we still found notable exceptions where (C + G)-rich repeats were dominant in specific genomic locations, including AGAGG and ACTGG in introns or intergenic regions of Trub, Csem and Okis and ACTGC in exons of Eluc (Tables 7, 8, 9). Although AGAGG repeats in introns and exons were relatively abundant in Csem, it was also the only species that lacked this repeat in intergenic regions in this study (Supplement Table 1). We also found that the CpG-containing repeats were present in the top 50% of pentanucleotide repeats, including (ATACG) n or (CCCGG) n tracts in intronic regions of Eluc and Locu, (CCCGG) n, (AATCG) n or (ACCGG) n tracts in exonic regions of Trub, Amex and Pret, and (ATACG) n or (ACCGG) n tracts in intergenic regions of Eluc and Pret (Tables 7, 8, 9).

### Hexanucleotide repeats

Hexanucleotide repeats were the least numerous in specific genomic regions, except for the exons of Trub (Table 3). In exonic and intronic regions, a dominance of (C + G)-rich repeats was found in the majority of the genomes (Tables 7 and 8). The repeat motifs present in intergenic regions were highly variable and relatively (A + T)-rich (Table 9). Except for in Olat, Onil, Ccar, Okis and Okis, the CpG-containing repeats were common in the top 50% of hexanucleotide repeats in intronic and exonic regions, and half of species had CpG-containing in the top 50% of hexanucleotide repeats in intergenic regions (Tables 7, 8, 9). A few telomere-like repeats were found in introns or intergenic regions, excluding Pret. However, the (AATCCC) n and (AACCCT) n tracts were observed in exonic regions of Trub and Omyk, respectively (Table 8).

### Iteration number and length distribution of microsatellites in fish genomes

**Fig. 1:**
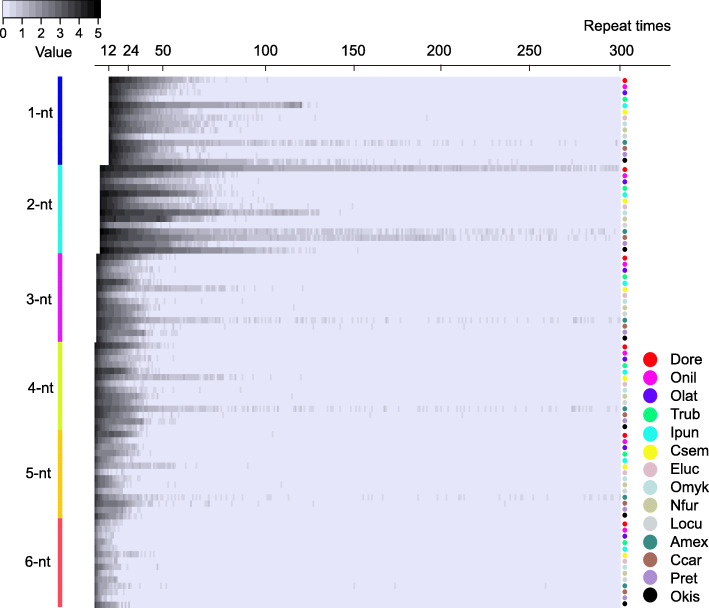
Heat map of the microsatellite distribution frequency of different motif length (1–6 nt) based on the iteration number among 14 fish genomes

Iteration number and length of microsatellites are both important factors determining microsatellite mutation rates, and it could be extremely important not only for genomic stability, but also with regard to the evolution of additional genomic features such as codon usage. To assess expandability of the repeats, iteration number of microsatellites was plotted against microsatellite length of various quantity intervals: <20, 20–50, 50–100, 100–200, 200–300, and >300 (Fig. 1). The details of all iteration numbers and densities of microsatellites in fish genomes are given in the Supplement Table 2. Usually, the frequency of microsatellites has a tendency to converge to a small iteration number. In other words, short microsatellites were observed more frequently in the fish genomes than long microsatellites. When the iteration number was less than 20, the repeat tracts varying motif lengths from mono- to hexa-nucleotide (1–6 nt) comprised more than 83.93, 67.22, 90.38, 88.93, 92.58 and 90.42%, respectively (Fig. 1 and Supplement Table 2). However, a few special microsatellites were found where the iteration number exceeded 300, for example 1-nt microsatellites in Csem, Eluc, Amex, Ccar and Okis, 2-nt microsatellites in Drer, Csem, Eluc, Nfur, Amex, Ccar and Okis, 3-nt microsatellites in Nfur, Amex, Ccar, 4-nt microsatellites in Drer, Csem, Nfur, Amex and Ccar, 5-nt microsatellites in Amex and Ccar, and 6-nt microsatellites in Csem, Amex and Ccar (Supplement Table 2).

## Discussion

In this study, we examined the microsatellites composed of motifs 1–6 bp long in the entire genomes of 14 fish species and analyzed their distribution and frequency in different genomic regions. Microsatellite occurrence significantly differed with the coverage degree varying from 0.18 to 5.29%. Comparison of microsatellite repeat occurrence in the genomes of humans (3%) [6], primates (0.83–0.88%) [72–74], birds (0.13–0.49%) [88], plants and fungi (0.04–0.15%) [75, 76, 80, 89, 90], with our data indicates that microsatellite occurrence differs between different species and this might be a general phenomenon across taxa [33]. In fact, differences might even occur between closely related species as humans and chimpanzees [91], and within the genus of *Drosophila* [92, 93]*.*

Another clear trend to emerge from this analysis was that the observed dependence of microsatellite abundance on repeated unit length and iteration number was very much biased from the expected trend of gradual decrease, which was consistent with a previous study [36]. Our research also indicated that microsatellite density is not strictly positively correlated with genome size. Although it was well known that the microsatellite density generally correlates positively with genome size [26, 36, 94], our contradictory results have been found in other studies [72, 83, 88, 95]. Overall, the comparative analysis of microsatellites indicated that there was great variation of microsatellite content across the 14 fish species. This might be indicative that differential selective constraints may play an important role in microsatellite evolution and result in the accumulated preference for different microsatellite types (Saeed2016&Ellegren2004& Schlötterer2000).

During genome evolution, microsatellite repeats mutation may provide a molecular mechanism for faster adaptation to environmental stress by increasing the quantities of DNA and providing the raw materials for adaptive evolution of organisms. Generally, microsatellite instability of dinucleotide repeats is higher than trinucleotide, tetranucleotide and pentanucleotide repeats [96]. In other words, the mutation rate of microsatellite dependence on repeated unit length is biased from the trend of gradual decrease. This could explain the high numbers of mono−/di-nucleotide motif microsatellites and the low numbers of penta−/hexa-nucleotide motif microsatellites in the genomes. We should note that the frequency of tetranucleotide repeats was more than trinucleotide repeats in most of the 14 genomes. However, there was a trend that trinucleotide repeats were more frequent than tetranucleotide repeats in exonic regions, and less than tetranucleotide repeats in intronic and intergenic regions of most genomes. We suggest that the lower number of trinucleotide repeats cannot only be explained by conservation since they attribute triplet codes to form parts of genes. However, there may be a mechanism (e.g. mismatch repair system) in the exonic regions to maintain the higher number of trinucleotide repeats.

As is evident from Tables 2, 3, 4, 5, 6, 7, 8 and 9, poly(A/T) tracts were more common than poly(C/G) tracts in these genomes. Poly(A/T) tracts were particularly common in exonic and intergenic regions, but this was opposite in intronic regions of some taxa (e.g., Trub, Omyk and Okis) and this has also been observed in the human genome [6]. The higher frequency of poly(A) tracts can be attributed to the re-integration of processed genes into the genome from mRNA with an attached poly(A) tail, while poly(C/G) are not part of this integrative mechanism. An alternative explanation is that a long A-rich tail is known to be necessary for the universal retrotransposon in eukaryotic genomes, such as *Alu*, LINE-1 and L1 retrotransposons [97–99]. Meanwhile, the formation of pseudogenes may attribute to this higher proportion of (A + T)-rich repeats [36, 100]. However, the mutation mechanism of microsatellite DNA provides a basis for this phenomenon. The variable frequencies of poly(A) and poly(C) could be due to the difference in stability between (GC) n and (AT) n repeats. (GC) n repeats are more stable than (AT) n repeats and hence it would be more difficult for the poly(C) sequences to slip during replication during the evolution of microsatellite DNA [6, 95, 101]. In the intronic regions, the higher than expected frequencies of poly(C/G) tracts in some species may be due to duplication events of key DNA sequences during evolution or the integrity of chromosomes may depend on a higher order DNA sequence organization that includes the presence of poly(C/G) tracts [102].

In the case of dimeric repeats, we found (AC) n tract was common and the (GC) n tract was rare. Assuming that, on the microsatellite DNA stability, (GC)-rich regions are relatively stable, there is less replication slippage generating the repeated motifs of microsatellites [103]. On a genomic scale, microsatellite sequences are presumably at equilibrium, where (AC) n or (AG) n repeats should be more abundant than (AT) n or n repeats. However, we found the opposite distribution of microsatellite motifs in the genome of Amex. We suggest that there is interspecific variation in the mechanisms of mutation or repair of specific motifs [63] or there might be variation in the selective constraints that are associated with different microsatellite motifs [33].

Compared to other microsatellite motifs, the trinucleotide repeat undergoes strict regulation under evolutionary stress. While the (AAT) n tracts were common in intronic and intergenic regions of the fish genomes, (AGG) n tracts were typically more numerous than other repeat types in exons. Therefore, different genome fractions may characterize different microsatellite abundances resulting from the functions of genome evolution and selective constraints [104]. Combined with the above, inconsistent distribution patterns where (ACT) n tracts were numerous in intergenic regions of Okis and (AAT) n tracts were common in exons of Drer and Ipun indicated that the distribution of microsatellites reflected the bias of the base composition in the genomes fractions. Other biases, such as the (CCG) n tracts in Trub and the (ACC) n tracts in Ccar, suggest that selective forces probably play various roles in specific genomes and differ from each other in a species-specific manner [36].

It should be noted that we found extremely rare (CCG) n and (ACG) n repeats in these genomes. A reasonable explanation for this rarity is the presence of the highly mutable CpG dinucleotide within the motif. Rarity of CpG is almost certainly a consequence of the methylation. In vertebrate genomes, a CpG-containing island occurs at about one-fifth of the expected frequency [105, 106] because between 60 and 90% of CpGs are methylated at the 5 position on the cytosine ring and there is a failure of the DNA repair mechanism to recognize deamination of 5-methylcytosine to produce thymine [107, 108]. However, experiments have shown that clusters of non-methylated CpG may attribute to the lack of CpG suppression in the HTF islands, where an approximate 1% DNA fraction accounted for the total genome from a variety of vertebrates [109, 110]. The HTF fraction is extremely rich in cleavable sites for mCpG-sensitive restriction enzymes and sequences chosen at random from the HTF fraction belong to islands of DNA several hundred base pairs long that contain CpG at more than 10 times its density in bulk DNA. This would help to explain the phenomenon that (ACG) n or (CCG) n tracts were abundant in introns of all fishes, in contrast to the rarity or absence of this motif in intergenic regions. An alternative explanation is that a specific mechanism exists to maintain the observed level of CpG-containing repeats in introns. The role of cytosine methylation in histone deacetylation, chromatin remodeling, and gene silencing may account for this phenomenon [111].

In the tetranucleotide microsatellites, the (AAAB) n tracts (B denotes any base other than A) seem to be more common, followed by 25% G + C content, and then 75% G + C content and 100% G + C content. Previous studies have indicated that DNA sequence composition could have a profound influence on microsatellite incidence [26, 33]. Kristitin et al. (2002) suggested that the G + C content of microsatellites might have influenced the mutation rate because the tetranucleotide repeats with 25% G + C content were not statistically different from each other, but each was significantly different from the repeats with 50% G + C content [112]. Meanwhile, the attribution of selective forces and DNA mismatch repair system for the distribution patterns could not be ignored, because of several exceptions observed in our study, for example (ACAG) n tracts were abundant in Omky and Okis.

The longer microsatellites (5–6 nt) have an advantage of being more polymorphic than the shorter ones (1–4 nt), as mutation rates generally increase with an increase in the number of repeat units [33, 113]. The significant differences in the repeat types and motif length of microsatellites between studied fish species seems to be due to their genome-specific characteristics. In conclusion, though it remains unclear why certain repeat motifs are more common than others, or the reason they vary so much between different fish species, several observations presented here suggest that individual genomes and genome-specific regions may be characterized by unique microsatellite profiles. This was also supported by the reports of taxon-specific repeats or genome-specific region repeats [6, 36]. The study of microsatellites may help us understand numerous aspects of genome organization and functions.

## Supplementary Information

**Additional file 1: Supplement Table 1.** Total numbers of different microsatellite repeats in 14 fish species.

**Additional file 2: Supplement Table 2.** Total numbers of Mono-, Di-, Tri- Tetra-, Penta-, and Hexanucleotide repeats in 14 fish species.

## Data Availability

Genome data are available using the links provided by the Ensembl team (*Astyanax mexicanus*: http://ftp.ensembl.org/pub/release-103/fasta/astyanax_mexicanus/dna/; *Danio rerio*: http://ftp.ensembl.org/pub/release-103/fasta/danio_rerio/dna/; *Cynoglossus semilaevis*: http://ftp.ensembl.org/pub/release-103/fasta/cynoglossus_semilaevis/dna/; *Esox Lucius*: http://ftp.ensembl.org/pub/release-103/fasta/esox_lucius/dna/; *Ictalurus punctatus*: http://ftp.ensembl.org/pub/release-103/fasta/ictalurus_punctatus/dna/; *Lepisosteus oculatus*: http://ftp.ensembl.org/pub/release-103/fasta/lepisosteus_oculatus/dna/; *Oryzias latipes*: http://ftp.ensembl.org/pub/release-103/fasta/oryzias_latipes_hni/dna/; *Oreochromis niloticus*: http://ftp.ensembl.org/pub/release-103/fasta/oreochromis_niloticus/dna/; *Poecilia reticulate*: http://ftp.ensembl.org/pub/release-103/fasta/poecilia_reticulata/dna/; *Takifugu rubripes*: http://ftp.ensembl.org/pub/release-103/fasta/takifugu_rubripes/dna/), and NCBI team (*Cyprinus carpio*: https://ftp.ncbi.nlm.nih.gov/genomes/all/GCF/000/951/615/GCF_000951615.1_common_carp_genome/; *Nothobranchius furzeri*: https://ftp.ncbi.nlm.nih.gov/genomes/all/GCF/001/465/895/GCF_001465895.1_Nfu_20140520/; *Oncorhynchus kisutch*: https://ftp.ncbi.nlm.nih.gov/genomes/all/GCF/002/021/735/GCF_002021735.2_Okis_V2/; *Oncorhynchus mykiss*: https://ftp.ncbi.nlm.nih.gov/genomes/all/GCF/013/265/735/GCF_013265735.2_USDA_OmykA_1.1/).
